# Q Neuron-Induced Hypothermia Promotes Functional Recovery and Suppresses Neuroinflammation after Brain Injury

**DOI:** 10.1523/JNEUROSCI.1035-25.2025

**Published:** 2025-10-13

**Authors:** Lisa Sakurai, Ryusuke Yoshimoto, Shingo Soya, Takeshi Sakurai

**Affiliations:** ^1^Institute of Medicine, University of Tsukuba, Tsukuba, Ibaraki 305-8575, Japan; ^2^International Institute for Integrative Sleep Medicine (WPI-IIIS), Tsukuba Institute for Advanced Research (TIAR), University of Tsukuba, Tsukuba, Ibaraki 305-8575, Japan; ^3^ Frontier Medical Science Degree Program, Graduate School of Comprehensive Human Sciences, University of Tsukuba, Tsukuba, Ibaraki 305-8577, Japan; ^4^ Humanics Degree Program, University of Tsukuba, Tsukuba, Ibaraki 305-8577, Japan; ^5^Life Science Center for Tsukuba Advanced Research Alliance, University of Tsukuba, Tsukuba, Ibaraki 305-8577, Japan

**Keywords:** astrocyte, brain injury, microglia, Q neuron-induced hypothermic/hypometabolic states

## Abstract

Traumatic brain injury (TBI) triggers a cascade of secondary pathologies—such as neuroinflammation and glial activation—contributing to progressive neuronal loss and hindering functional recovery. While therapeutic hypothermia has shown neuroprotective potential, its clinical application is limited by systemic complications. Recent discoveries have identified hypothalamic Q neurons, whose activation induces a reversible, hibernation-like hypothermic state, termed Q neuron-induced hypothermic/hypometabolic states (QIH), without the need for external cooling. However, whether QIH can mitigate brain injury remains unknown. In this study, we examined the therapeutic effects of QIH following acute brain injury in male mice. Using a dorsal striatal stab injury model, we found that QIH-treated mice displayed significantly improved motor performance and grip strength compared with controls. Histological analyses revealed enhanced neuronal survival in the perilesional striatum, accompanied by markedly reduced astrocytic gliosis and microglial accumulation at the injury site. To investigate the mechanisms underlying these improvements, we employed a medial prefrontal cortex injury model and observed that QIH robustly suppressed astrocytic and microglial activation, as indicated by reduced GFAP and Iba1 expression. Additionally, QIH decreased the number of CD16/32- and CD68-positive microglia and downregulated iNOS expression, suggesting that QIH dampens both oxidative and phagocytic inflammatory responses. Morphometric analysis further revealed a shift toward ramified and rod-shaped microglia, phenotypes associated with neuroprotection. Our findings demonstrate that QIH ameliorates early neuroinflammation, preserves neuronal integrity, and promotes functional recovery following brain injury. These results highlight QIH as a novel and physiologically grounded neuroprotective strategy that may overcome the limitations of conventional hypothermia-based interventions.

## Significance Statement

Traumatic brain injury (TBI) often leads to long-term neurological impairments due to glial activation and neuroinflammation. Although therapeutic hypothermia can reduce secondary damage, its clinical use is limited by systemic side effects. Here, we demonstrate that a hibernation-like state induced by hypothalamic Q neurons—Q neuron-induced hypothermic/hypometabolic states (QIH)—improves motor function, enhances neuronal survival, and suppresses early neuroinflammatory responses in mouse models of brain injury. QIH attenuated astrocytic and microglial activation and promoted the emergence of neuroprotective microglial morphologies. These results suggest that QIH is a promising and physiologically regulated neuroprotective strategy. Unlike traditional hypothermia, QIH avoids external cooling, offering a potentially safer and more practical approach to TBI treatment.

## Introduction

Traumatic brain injury (TBI) is a major public health concern that frequently leads to persistent neurological impairments and functional disability (Bramlett and Dietrich, 2015). Caused by external mechanical forces, such as impact, penetration, or rapid acceleration–deceleration, TBI initiates both immediate and delayed pathological responses. The primary injury involves neuronal and glial cell loss, axonal damage, cerebral edema, and disruption of the blood–brain barrier (Puntambekar et al., 2018; Mira et al., 2021). These acute events subsequently trigger secondary cascades—including oxidative stress, neuroinflammation, and metabolic dysregulation—that exacerbate tissue damage and impair recovery.

A hallmark of secondary injury is the rapid activation of glial cells—microglia and astrocytes—which mediate inflammatory responses and participate in tissue repair (Mira et al., 2021). Microglia adopt amoeboid morphologies, migrate to the lesion site, and secrete proinflammatory cytokines (Donat et al., 2017), while astrocytes undergo reactive gliosis, marked by hypertrophy and upregulation of intermediate filaments such as GFAP (Robel et al., 2011; Dimou and Götz, 2014). Although these glial responses can be protective, their sustained activation contributes to neuronal loss, impedes regeneration, and worsens long-term outcomes (Donat et al., 2017; Guttenplan et al., 2021).

Therapeutic hypothermia has shown potential for mitigating secondary damage by reducing inflammation and metabolic demand (Andresen et al., 2015). However, conventional cooling methods are associated with systemic complications such as insulin resistance, coagulopathy, and cardiac arrhythmias, limiting their clinical application (Lewis et al., 2017).

Recent advances in neurobiology have identified a distinct population of hypothalamic neurons—termed Q neurons—whose activation induces a reversible, hibernation-like hypothermic and hypometabolic state, referred to as Q neuron-induced hypothermic/hypometabolic states (QIH; Takahashi et al., 2020). Unlike conventional hypothermia, QIH does not require external cooling and achieves physiological downregulation through endogenous mechanisms. While QIH has been shown to lower body temperature and metabolic rate, its therapeutic potential in the context of brain injury has not been explored.

Here, we investigate whether QIH can promote functional recovery and attenuate neuroinflammation after acute brain injury in mice. We employed a chemogenetic approach to induce QIH using *Qrfp-iCre;Rosa26^dreaddm3^* mice (hereafter Qrfp-M3 mice), which enable systemic activation of Q neurons via clozapine-*N*-oxide (CNO) without the need for intracranial viral vector injection. This model avoids local inflammation and potential confounds associated with surgical viral vector delivery and provides a more controlled platform for evaluating therapeutic outcomes.

Our findings reveal that QIH enhances motor recovery, preserves neuronal integrity, and suppresses early glial activation following striatal and cortical injuries. These results support QIH as a novel, physiologically regulated neuroprotective strategy with the potential to overcome limitations of conventional hypothermia in TBI management.

## Materials and Methods

### Animals

All animal experiments were conducted at the International Institute for Integrative Sleep Medicine, University of Tsukuba, in accordance with the institute's animal experimentation guidelines. The experimental protocols were approved by the Animal Experimentation Committee (Approval Number 24-065) and adhered to the guidelines of the US National Institutes of Health (NIH). Mice were housed under controlled conditions (23°C, 50% humidity, 12 h light/dark cycle) with *ad libitum* access to food and water. Experiments were performed using adult male C57BL/6J wild-type mice (Charles River Laboratories, ID#000664) and *Qrfp-iCre;Rosa26^dreaddm3^*mice (Qrfp-M3 mice; Takahashi et al., 2020), aged 6–12 weeks and weighing 23–30 g. All mice were group-housed (four per cage) without isolation. Only male mice were used in this study.

### Chemogenetic induction of QIH

QIH was induced in Qrfp-M3 mice using the DREADD system. CNO (Abcam, ab141704) was dissolved in saline (500 μg/ml) and stored at −20°C. For QIH induction, CNO was intraperitoneally injected at a dose of 6.5 mg/kg. Because Qrfp-M3 mice exhibit weaker and shorter QIH responses than Q-hM3Dq mice (which require AAV delivery into the AVPe), supplemental doses of CNO (2.5–5 mg/kg) were administered as needed to maintain QIH.

Body temperature was continuously monitored using a thermal imaging camera (Nippon Avionics) inside a temperature-controlled chamber (HC-100, Shin Factory) set to 22.0°C. Mice were not shaved to avoid unnecessary stress, and measurements were taken at the interscapular region. Additional doses of CNO were given if body temperature exceeded 28°C during the designated QIH period.

### Induction of brain injury

Stereotaxic surgery was performed under isoflurane anesthesia (Pfizer). For striatal injury, a sterile syringe needle (23G × 1, 0.60 × 25 mm, NN-2325R) was inserted into the dorsal striatum at the following coordinates: anterior–posterior (AP) + 1.75 mm, medial–lateral (ML) + 0.74 mm, and dorsal–ventral (DV) −4.0 mm. The needle was held in place for 5 min and then slowly withdrawn over ∼1 min.

For cortical injury, a fiber-optic cannula (RWD Life Science, 200 μm core, 6 mm length) was stereotaxically inserted into the medial prefrontal cortex (mPFC) at AP +0.3 mm, ML +1.94 mm, and DV −4.0 mm. Although the mPFC is located near the midline, the left and right hemispheres are anatomically distinct, and clear laterality in glial responses was observed, supporting the use of unilateral injuries to evaluate regional differences.

To investigate molecular changes via quantitative polymerase chain reaction (qPCR), bilateral cortical injuries were induced by inserting syringe needles into the mPFC at AP ±0.3 mm, ML +1.94 mm, and DV −4.0 mm. Bilateral injury was chosen to obtain sufficient tissue for RNA extraction while minimizing the number of animals used, in accordance with animal welfare guidelines. The use of both cortical (mPFC) and subcortical (dorsal striatum) injury models allowed us to assess the generalizability of QIH effects across distinct brain regions with differing vulnerability and glial architecture.

### Behavioral tests

All behavioral tests were conducted under dim lighting conditions (<15 lux) and recorded using a CCD video camera. Behavioral data were analyzed using DeepLabCut (https://github.com/DeepLabCut/DeepLabCut) and the OpenCV (cv2) Python module.

#### Open-field test

Spontaneous locomotor activity was assessed in an open-field arena (40 × 40 cm), as previously described (Seibenhener and Wooten, 2015). Each mouse was placed in the center of the empty arena and recorded for 2 min and 15 s. Key parameters—including total distance traveled, maximum velocity, and average velocity—were calculated to evaluate motor function. The open-field test was conducted on postinjury days (POD) 3, 5, 10, and 15 to assess locomotor recovery over time.

To quantify gait-related movement, five 1 s video segments per mouse were extracted during periods of straight walking. The nose, hip, and tail base were tracked frame-by-frame to compute body segment angles. Temporal changes in these angles were fitted with cosine functions, and the amplitude of each fitted curve was used as an index of locomotor coordination.

#### Grip strength test

Forelimb and four-limb grip strength were evaluated on POD 20 using a Mouse/Rat Grip Strength Meter (GPM-101BV/GPM-101B/GPM-101V, Melquest), as previously reported (Takeshita et al., 2017). In the forelimb test, mice grasped a horizontal bar with their front paws, and the tail was gently pulled backward until the grip was released. In the four-limb test, mice held onto a vertical mesh grid using all four limbs.

Each mouse performed three consecutive trials for each test. Grip force was normalized by body weight (grams) and averaged across trials.

Following the final behavioral tests, mice were immediately perfusion-fixed for histological analyses.

### Immunohistochemistry

Mice were anesthetized with isoflurane and perfused transcardially with 10% sucrose solution, followed by 4% paraformaldehyde (PFA). Brains were postfixed in 4% PFA in phosphate-buffered saline (PBS) for 24 h and cryoprotected by immersion in 30% sucrose for 2 d. Brain sections (40 μm thick) were prepared using a cryostat (Leica Biosystems CM1860).

The sections were washed three times in PBS for 10 min each and incubated overnight at 4°C with primary antibodies in PBS containing 10% bovine serum albumin (Blocking One, Nacalai Tesque) and 0.3% Triton X-100. The primary antibodies used were goat anti-Iba1 (1:500; 011-27991, Fujifilm), rabbit anti-GFAP (1:2,000; HPA056030, Sigma-Aldrich), rabbit monoclonal anti-CD16 + CD32 antibody (1:500;EPR23501-203, Abcam), rat monoclonal anti-CD68 antibody (1:50; FA-11, Abcam), and mouse monoclonal anti-NeuN antibody(1:500; clone A60, MAB377, Sigma-Aldrich). Sections were then washed three times in PBS and incubated overnight at 4°C with secondary antibodies in PBS containing 10% bovine serum albumin and 0.3% Triton X-100. The secondary antibodies used were Alexa Fluor 488-conjugated donkey anti-goat IgG (H + L), cross-adsorbed (1:1,000; A11055, Invitrogen); Alexa Fluor Plus 594-conjugated donkey anti-rabbit IgG (H + L), highly cross-adsorbed (1:1,000; A32754, Invitrogen); Alexa Fluor Plus 488-conjugated donkey anti-mouse IgG (H + L), highly cross-adsorbed (1:1,000; A32766, Invitrogen); and Alexa Fluor Plus 488-conjugated donkey anti-rat IgG (H + L), highly cross-adsorbed (1:1,000; A48269, Invitrogen). Sections were washed in PBS, air-dried on subbed slides, and coverslipped using FluorSave reagent (Calbiochem). Brain sections were observed using a confocal laser microscope (SP8, Leica Biosystems) or a fluorescent microscope (THUNDER imaging system, Leica Biosystems).

### Quantitative reverse transcription polymerase chain reaction (RT-qPCR)

Mice were anesthetized with isoflurane to achieve deep anesthesia, confirmed by the absence of pain reflexes. The brain was promptly removed and immersed in ice-cold, half-frozen artificial cerebrospinal fluid to preserve its structural integrity. After a 3 min immersion, the cerebellum was removed, and the remaining brain tissue was sliced into 50 µm sections using a vibratome (VT1200S, Leica Biosystems). The mPFC region surrounding the injury site was dissected and placed in a 1.5 ml tube, immediately frozen in liquid nitrogen, and stored at −80°C for further processing.

Total RNA was extracted from the brain tissue around the injured area using the NucleoSpin RNA XS kit (TaKaRa Bio; Product Codes, 740902.50 and 740944). The quality and concentration of the extracted RNA were measured and recorded using NanoDrop One/OneC Microvolume UV-Vis Spectrophotometer (ND-ONE-W, Thermo Fisher Scientific).

For reverse transcription of RNA, we used the ReverTra Ace qPCR RT Kit (FSQ-101, TOYOBO) following the manufacturer's protocol. To denature secondary RNA structures, RNA samples were incubated at 65°C for 5 min and then rapidly cooled on ice for 30 s. The reverse transcription reaction was carried out at 37°C for 15 min, followed by inactivation and denaturation at 98°C for 5 min. The resulting complementary DNA was stored at 4°C until further use.

qPCR was performed using the THUNDERBIRD Probe qPCR Mix (QPS-101T, TOYOBO), optimized for high sensitivity and specificity. Specific primer-probe sets for IL-1β, IL-10, iNOS, and the internal control gene GAPDH were designed and synthesized. The primer sequences used were as follows:

IL-1β: forward, GACCTGTTCTTTGAAGTTGACG; reverse, CTCTTGTTGATGTGCTGCTG
IL-10: forward, TTGAATTCCCTGGGTGAGAAG; reverse, TCCACTGCCTTGCTCTTATTT
iNOS: forward, GGAATCTTGGAGCGAGTTGT; reverse, CCTCTTGTCTTTGACCCAGTAG

qPCR amplification was conducted using a CFX96 Real-Time PCR System (Bio-Rad Laboratories) under the following cycling conditions: initial denaturation at 95°C for 30 s, followed by 40 cycles of 95°C for 15 s, 55°C for 30 s, and 72°C for 30 s. Fluorescence was monitored at each cycle.

Gene expression levels were quantified using the comparative ΔCq method, with GAPDH as the internal control. Each reaction was performed with *N* = 8 biological replicates, and relative mRNA expression levels were calculated as 2^(−ΔCq)^. Data were analyzed using CFX Manager (Bio-Rad Laboratories) and presented as fold change relative to the control group.

### Analysis of cellular numbers and morphology

#### Cell counting

Glial cell numbers were quantified using ImageJ (Schneider et al., 2012) from brain sections containing injury sites. Cells were counted within a 500 × 500 µm area centered on the injury tip, and density was calculated as cells per square millimeter.

#### Morphological analysis

Microglial morphology was analyzed using scikit-learn, scikit-image, and napari in the Python module. *Z*-stack images were processed into 2D using maximum intensity projection, converted to grayscale, and background-corrected using a Gaussian filter, background subtraction, and fast Fourier transform for contour extraction. Nonoverlapping single cells (20 per section) were isolated and binarized using a threshold calculated by Otsu's method (Lai and Rosin, 2014).

#### Morphological parameters

The following parameters were calculated (Fernández-Arjona et al., 2017; Green et al., 2022):

Fractal Dimension and Lacunarity: Structural complexity and heterogeneity were assessed using the box-counting method.

Convex Hull Analysis: Measures of the cell and convex hull area, perimeter, and circularity. Density and roughness were calculated from these values.

Sholl Analysis: Dendritic complexity was assessed by counting intersections with concentric circles (D A Sholl, 1953).

Process Length and Straightness Index: A skeletonized binary image was used to calculate total and mean process lengths, with the straightness index defined as the ratio of Euclidean to actual path length.

Cell Body Area: A polygon was created for the cell body, and its area was measured using the Shoelace theorem and then converted to square micrometer.

#### Microglia categorization

The weights of morphological parameters were determined using the multimodality index (MMI), and features associated with the amoeboid type were selected (Table 1; Fernández-Arjona et al., 2017; Vidal-Itriago et al., 2022). The amoeboid index was defined as the sum of these scaled features. Similarly, the ramified index was calculated using features characteristic of the ramified type. Microglia classification was determined by comparing these indices, with each cell assigned to the predominant type based on the larger value.

**Table 1.** Morphological features enriched in amoeboid or ramified microglia identified by MMI analysis

Parameter	MMI	Morphology associated with increased values
Fractal dimension	0.347974	Ramified
lacunarity	0.50747	Ramified
Cell area (µm^2^)	0.464576	Amoeboid
Convex hull area (µm^2^)	0.424609	Ramified
Density	0.513855	Amoeboid
Cell perimeter (µm)	0.430981	Ramified
Convex hull perimeter (µm)	0.294781	Ramified
Roughness	0.446389	Ramified
Convex hull circularity	0.543737	Amoeboid
Cell circularity	0.600643	Amoeboid
Total_Process_Length	0.430398	Ramified
Max_Process_Legth	0.314099	Ramified
Mean_Process_Length	0.207123	Ramified
Sholl_Max_Counts	0.378631	Ramified
Straightness	0.286425	Ramified
Cell body area (μm^2^)	0.582923	Amoeboid

#### Cluster and principal component analysis

Hierarchical clustering analysis: Microglia were grouped based on morphometric parameters using the Scipy module in Python. Cell distances or similarities were calculated using Euclidean distance with Ward's method (Ward, 1963). Morphological parameters with an MMI ≥0.5 were selected for clustering into distinct cell types.

Cluster selection: The optimal number of clusters was estimated using the silhouette score (Rousseeuw, 1987). A silhouette score closer to 1 indicates higher cluster quality, and the final number of clusters was determined based on the maximized the silhouette score.

Principal component analysis (PCA): PCA was performed to identify key morphological variations, ensuring that the first two principal components explained >70% of the variance. Cell distributions were visualized based on hierarchical clustering and treatment conditions.

#### Quantification of glial coverage at the injury site of the striatum

Due to the high density and morphological complexity of glial cells at 20 d postinjury, reliable cell counting was not feasible. Instead, we quantified the area occupied by GFAP- and Iba1-positive signals as an index of astrocytic and microglial activation, respectively.

Images were acquired from brain sections containing the injury site using a fluorescence microscope (THUNDER imaging system, Leica Biosystems) under constant exposure settings. A standardized 500 × 500 µm region centered on the lesion core was defined for each image. The ImageJ software (NIH) was used to manually threshold the GFAP and Iba1 channels, and the total immunopositive area was calculated as a percentage of the defined region. All analyses were performed blinded to treatment condition.

### Statistical analysis

Data are presented as mean ± SEM unless otherwise noted. Statistical analyses were performed using repeated-measures ANOVA for the open-field test and amoeboid ratio. *p* values <0.05 were considered statistically significant. For comparisons between two groups, an unpaired two-tailed Student’s *t* test was used. We report exact test statistics and degrees of freedom for all tests in accordance with JNeurosci policy.

### Code availability

The code used to perform all analyses in this study is available at https://github.com/Yoshimoto-1/program-paper-TBI-in-QIH.git.

## Results

### QIH facilitates functional recovery following striatal injury

**Figure 1.**
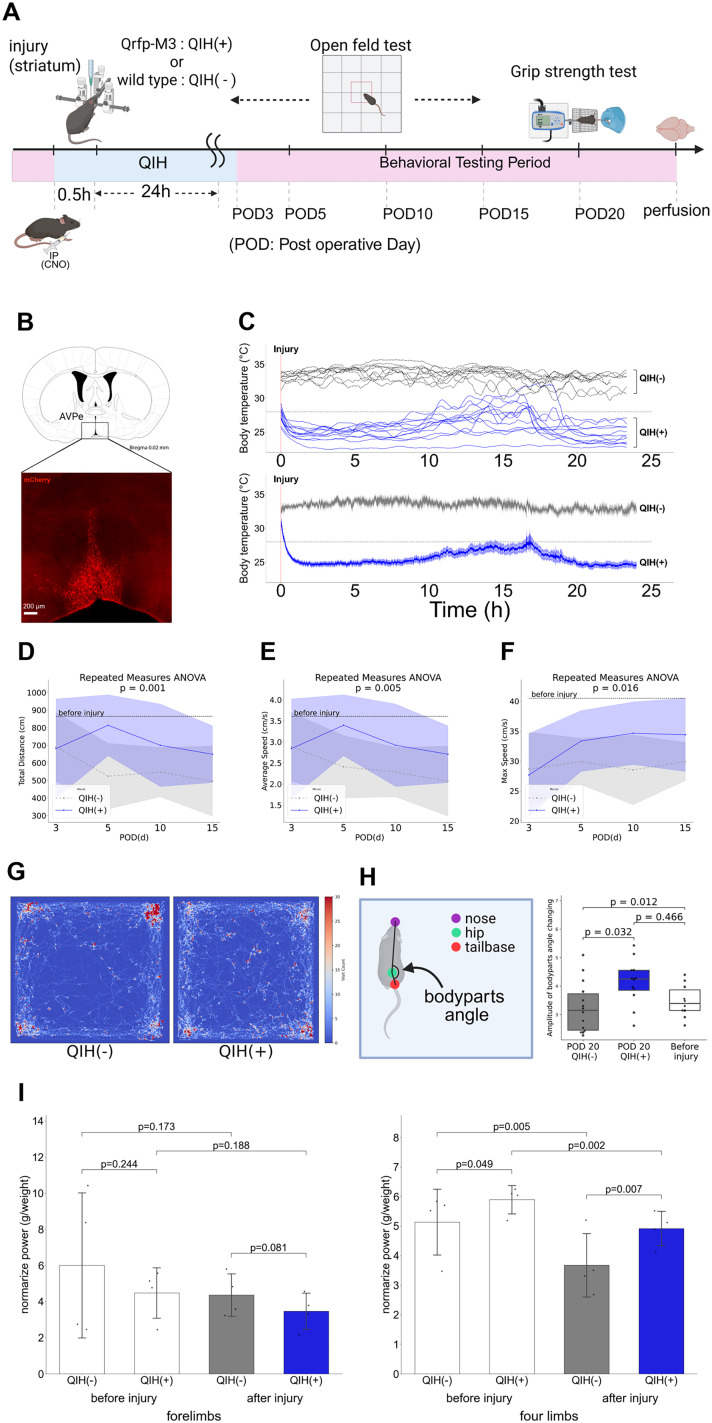
QIH promotes functional recovery following striatal injury. ***A***, Experimental timeline. A unilateral dorsal striatal stab injury was introduced, followed 30 min later by intraperitoneal injection of CNO (6.5 mg/kg) to induce QIH. Behavioral assessments consisted of open-field tests conducted on POD 3, 5, 10, and 15 and grip strength testing performed on POD 20. ***B***, Representative image of hM3Dq-mCherry expression in the anteroventral periventricular nucleus (AVPe) of Qrfp-M3 mouse, confirming selective targeting of Q neurons (scale bar, 200 μm). ***C***, Body temperature profiles following QIH induction. Top, Temporal changes in body temperature of individual mice. Bottom, Group averages ± SEM. (*N* = 12 per group). In the QIH(+) group (blue), a marked drop in body temperature was induced, and sustained hypothermia was maintained for over 24 h with additional CNO injections as needed. In contrast, no change in body temperature was observed in the QIH(−) group (gray). ***D–F***, Open-field test performance. Total distance traveled (***D***), average speed (***E***), and maximum speed (***F***) were significantly higher in QIH-treated mice at POD 5, 10, and 15 (repeated-measures ANOVA, total distance, *F*_(3,66)_ = 6.42; *p* = 0.0007; average speed, *F*_(3,66)_ = 5.98; *p* = 0.0011; maximum speed, *F*_(3,66)_ = 4.87; *p* = 0.0039; *N* = 12 per group). Dotted black lines represent baseline values obtained prior to injury from the same animals [QIH(−), *N* = 4; QIH(+), *N* = 4; total *N* = 8] and serve as reference values from uninjured mice. ***G***, Heatmaps representing positional occupancy in the open-field arena on POD 15. QIH-treated mice explored the arena more broadly, whereas control mice predominantly remained near the corners. ***H***, Gait analysis. The amplitude of angular fluctuation was greater in QIH(+) mice [unpaired two-tailed Student's *t* test: QIH(+) vs QIH(−), *t*_(24)_ = 2.71; *p* = 0.012; *N* = 12 vs 14]. Preinjury baseline comparisons: QIH(+) before versus after, *t*_(20)_ = 2.31; *p* = 0.032; QIH(−) before versus after, *t*_(22)_ = −0.74; *p* = 0.466. ***I***, The grip strength test on POD 20. **Forelimb**, No significant difference (unpaired two-tailed Student's *t* test, *t*_(22)_ = −1.83; *p* = 0.081). **Four-limb**, Significantly greater in QIH(+) versus QIH(−) (*t*_(22)_ = 3.00; *p* = 0.007).

To evaluate whether QIH promotes functional recovery after brain injury, we performed behavioral analysis in mice subjected to dorsal striatal stab injury. QIH was induced using Qrfp-M3 mice, which express hM3Dq selectively in *Qrfp*-positive neurons, allowing noninvasive activation of Q neurons via intraperitoneal injection of CNO (Fig. 1*A*). hM3Dq-mCherry expression was detected in the AVPe, in agreement with previous reports (Takahashi et al., 2020; Fig. 1*B*). As QIH induction in this model is less robust than in AAV-injected Q-hM3Dq mice (Takahashi et al., 2020), repeated CNO administration was used to maintain the hypothermic state. QIH was successfully sustained for over 24 h using a temperature-controlled chamber, whereas wild-type mice showed no temperature change in response to CNO (Fig. 1*C*).

Motor performance was assessed using open-field tests on POD 3, 5, 10, and 15. Both QIH(−) and QIH(+) groups initially exhibited impaired locomotion, but QIH(+) mice showed progressive improvement over time. By POD 10 and 15, they traveled significantly longer distances and moved at faster velocities compared with the QIH(−) group (Fig. 1*D*–*F*; Movie 1).

**Movie 1.** Open-field locomotion in QIH(−) and QIH(+) mice. Representative videos showing locomotion after TBI. The QIH(−) mouse (left) displays impaired movement, while the QIH(+) mouse (right) shows improved coordination and mobility. [View online]

In addition to reduced mobility, the QIH(−) group also displayed behavioral signs of anxiety or hesitancy, tending to remain in the corners of the open field more than the QIH(+) group (Fig. 1*G*). Gait analysis based on body segment angles—measured from the nose, hip, and tail base—revealed that the QIH(−) group walked with relatively constant angles between these points, suggesting a stiff or constrained locomotor pattern. In contrast, the QIH(+) group and uninjured group exhibited greater amplitude in angular fluctuations, indicating more dynamic and coordinated movement (Fig. 1*H*).

On POD 20, the QIH(+) group demonstrated significantly higher four-limb grip strength compared with the QIH(−) group (Fig. 1*I*).

Because QIH could potentially influence locomotor behavior or induce subtle gait abnormalities independent of injury, we also examined uninjured QIH(−) and QIH(+) mice. No significant differences were observed in open-field performance or grip strength (Fig. S1), indicating that the improved motor recovery observed in injured QIH(+) mice was not attributable to baseline effects of QIH itself. These results suggest that QIH promotes motor recovery after striatal injury by enhancing both activity levels and movement coordination.

### QIH preserves neuronal integrity near the injury site

**Figure 2.**
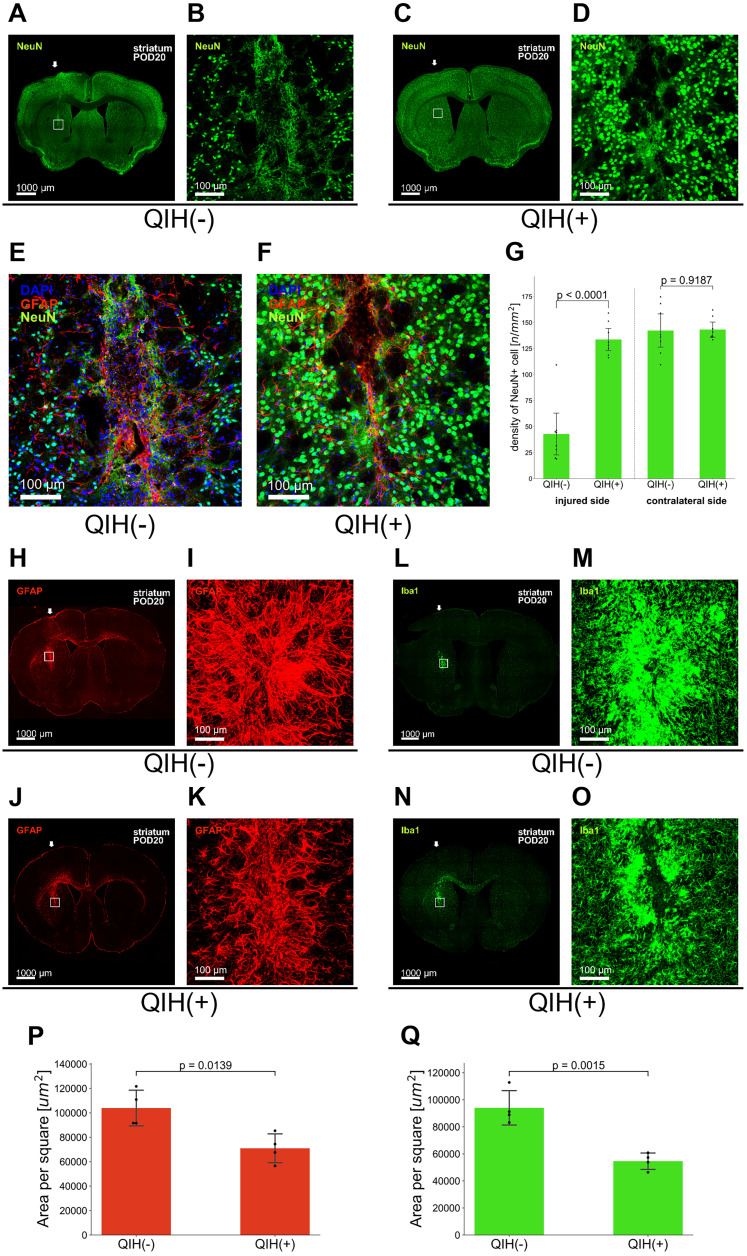
QIH enhances neuronal survival and modulates glial responses around the injury site in the striatum. ***A–D***, Representative immunofluorescence images of NeuN-positive neurons in coronal brain sections from the injured striatum in QIH(−) (***A, B***) and QIH(+) group (***C, D***) at 20 d after striatal injury. ***B*** and ***D*** show higher-magnification views of the boxed regions in ***A*** and ***C***, respectively. ***E, F***, High-magnification (100×) merged images of the perilesional striatum stained for NeuN (green), GFAP (red), and DAPI (blue) in QIH(−) (***E***) and QIH(+) groups (***F***). ***G***, NeuN+ density was higher in QIH(+) mice (unpaired two-tailed Student's *t* test, injured, *t*_(14)_ = −7.83; *p* < 0.0001; contralateral, *t*_(14)_ = −0.10; *p* = 0.919; *N* = 8 per group). ***H–K***, Representative images of GFAP immunoreactivity in the striatum surrounding the lesion site from the QIH(−) group (***H, I***) and QIH(+) group (***J, K***). Panels ***I*** and ***K*** are enlarged views of the corresponding boxed regions in panels ***H*** and ***J***. ***L–O***, Representative images of Iba1 immunoreactivity in the same region from the QIH(−) group (***L, M***) and QIH(+) group (***N, O***), with higher-magnification views shown in ***M*** and ***O***. ***P, Q***, Quantification of GFAP+ and Iba1+ areas. QIH(+) mice showed reduced GFAP and Iba1 immunoreactivity (unpaired two-tailed Student's *t* test, GFAP, *t*_(6)_ = 3.44; *p* = 0.014; Iba1, *t*_(6)_ = 5.49; *p* = 0.0015; *N* = 4 per group).

To examine the structural basis underlying the functional recovery observed in the QIH(+) group, we performed NeuN immunostaining to assess neuronal survival near the injury site in the striatum. On POD 20, the QIH(−) group showed a marked loss of NeuN-positive neurons in the perilesional region, consistent with secondary neuronal degeneration following injury (Fig. 2*A*,*B*,*E*). In contrast, the QIH(+) group exhibited significantly higher densities of NeuN-positive cells surrounding the lesion (Fig. 2*C*,*D*,*F*). Quantitative analysis confirmed a robust preservation of neuronal integrity in the QIH(+) group (Fig. 2*G*).

We next evaluated astrocytic and microglial responses in the same region at POD 20. GFAP and Iba1 immunostaining revealed visually reduced gliosis in the QIH(+) group compared with QIH(−) controls (Fig. 2*H*–*O*). To quantify these observations, we measured the immunopositive area for GFAP and Iba1 within a standardized 500 × 500 μm region surrounding the lesion. Both astrocytic and microglial signals were significantly lower in the QIH(+) group (Fig. 2*P*,*Q*), indicating that QIH suppresses chronic glial activation in the perilesional striatum.

Together, these findings demonstrate that QIH not only preserves neuronal structure but also attenuates chronic glial activation, suggesting that its neuroprotective effects extend beyond the acute phase and contribute to a sustained reduction in secondary neuroinflammatory processes.

**Figure 3.**
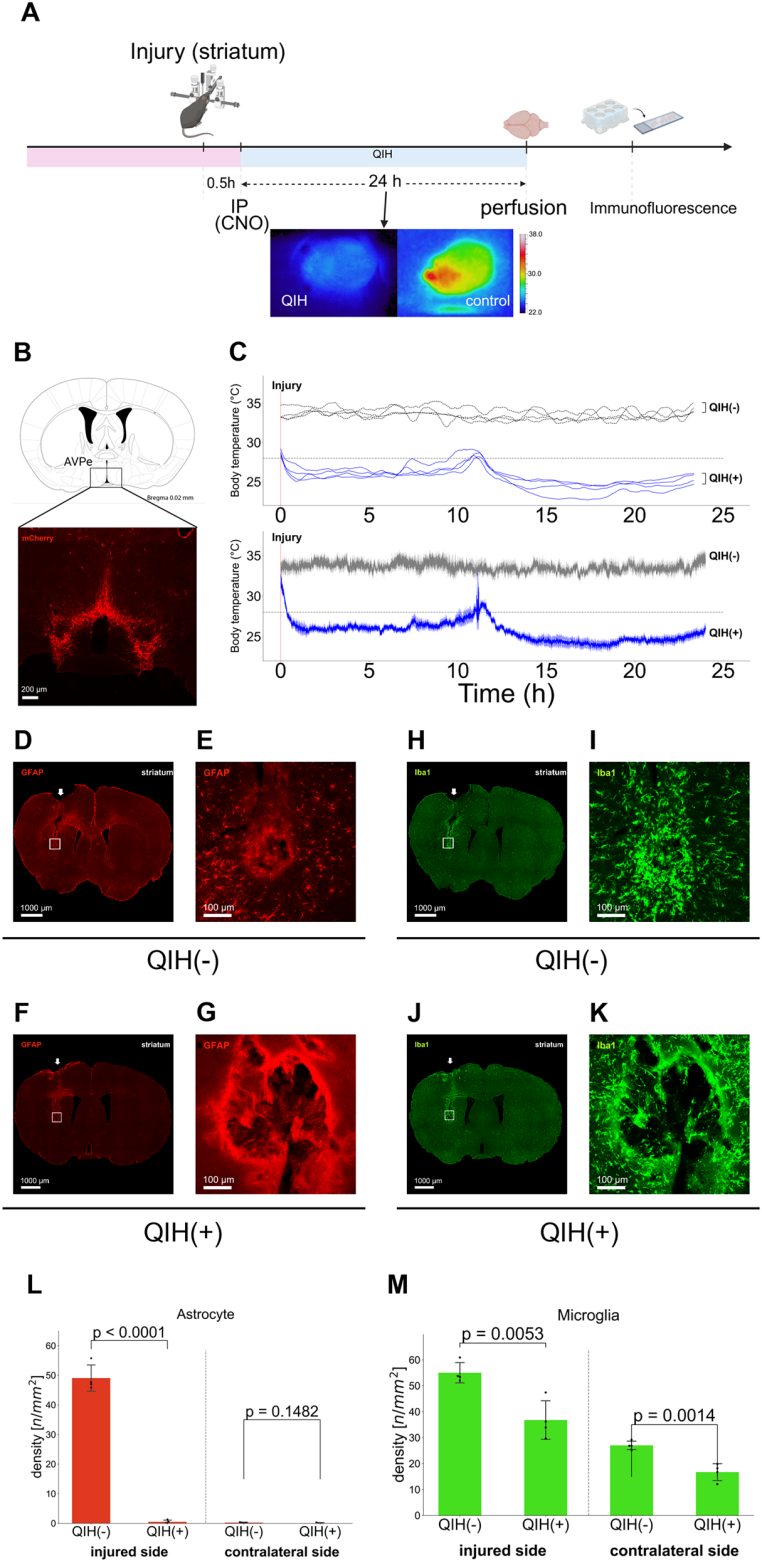
QIH suppresses astrocytic and microglial activation around the injury site in the striatum. ***A***, Experimental procedure. Stab injury was introduced 30 min before inducing QIH. Bottom panels, Representative thermographic images of experimental mice. ***B***, A representative image showing hM3Dq-mCherry expression in the AVPe of a Qrfp-M3 mouse. ***C***, Top panel, Temporal changes in the body temperature of individual mice. Bottom panel, Average ± SEM of body temperature values shown in the top panel (*N* = 4 per group). ***D–G***, GFAP-positive astrocytes around the lesion site in the striatum. Arrowheads indicate the insertion site of the needles. ***D, E***, QIH(−) group; (***F, G***) QIH(+) group. ***E, G***, Higher-magnification views of the boxed areas in ***D*** and ***F***, respectively. ***H–K***, Iba1-positive microglia around the lesion site in the striatum. ***H, I***, QIH(−) group; (***J, K***) QIH(+) group. ***I, K***, Higher-magnification views of the boxed areas in ***H*** and ***J***, respectively. ***L***, GFAP+ density was reduced ipsilaterally in QIH(+) mice (unpaired two-tailed Student's *t* test, *t*_(6)_ = 21.31; *p* < 0.0001), but not contralaterally (*t*_(6)_ = 1.66; *p* = 0.148). ***M***, Iba1+ density was reduced in both hemispheres (ipsilateral, *t*_(6)_ = 4.26; *p* = 0.005; contralateral, *t*_(6)_ = 5.56; *p* = 0.001).

Furthermore, to capture the early phase of glial activation, we also analyzed the tissue collected 24 h after striatal injury (Fig. 3*A*–*C*). GFAP immunostaining revealed a dense accumulation of reactive astrocytes around the lesion site in the QIH(−) group, whereas the QIH(+) group exhibited markedly reduced GFAP expression (Fig. 3*D*–*G*,*L*). Similarly, Iba1 immunostaining showed a significant reduction in microglial density in the injured cortex of QIH(+) mice compared with the QIH(−) group (Fig. 3*H*–*K*,*M*). A comparable decrease in microglia was also observed in the contralateral intact striatum, suggesting that QIH exerts a systemic suppressive effect on glial activation.

This early-phase analysis complements the chronic-phase findings at 20 d and reveals the temporal dynamics of astrocytic and microglial modulation under QIH treatment.

### QIH suppresses neuroinflammation: insights from a cortical injury model

**Figure 4.**
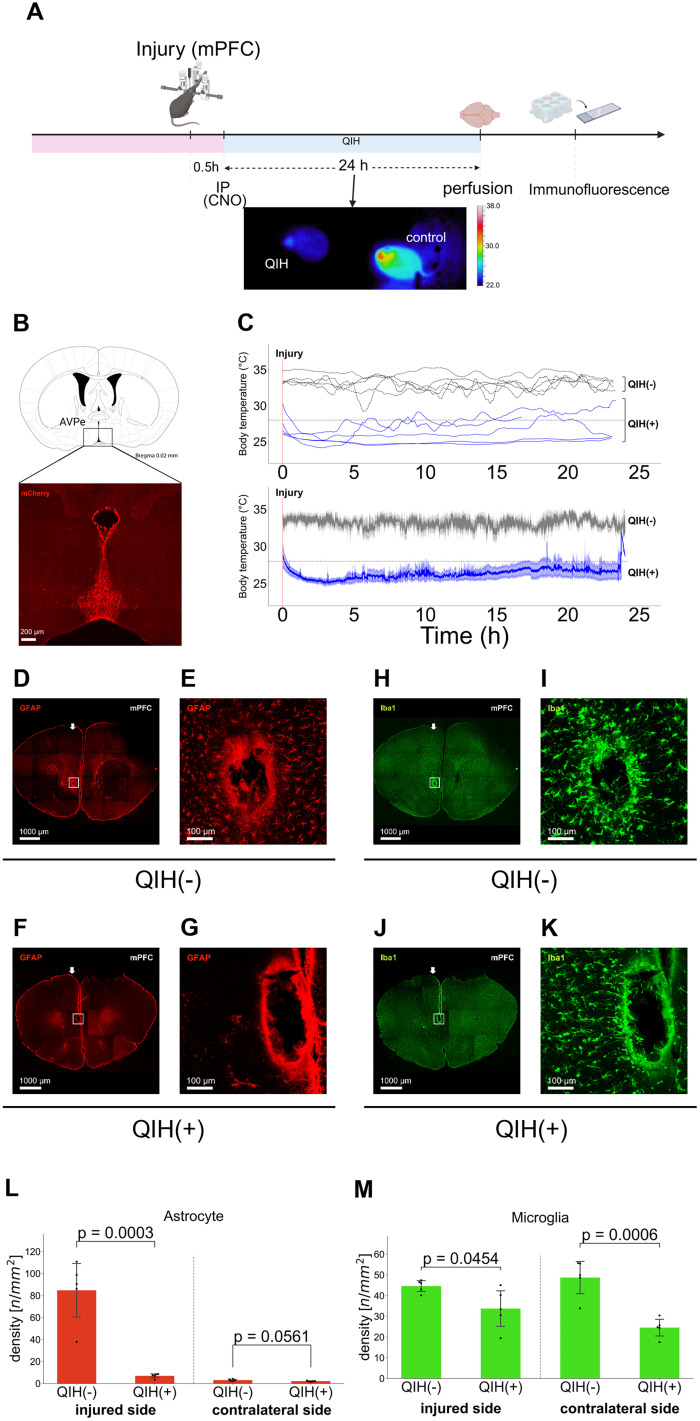
Effect of QIH on astrocytic and microglial responses around a stab injury in the mPFC in mice. ***A***, Experimental procedure. A stab injury was introduced 30 min before inducing QIH. Bottom panels, Representative thermographic images of experimental mice. ***B***, Representative image showing hM3Dq-mCherry expression in the AVPe of a Qrfp-M3 mouse. ***C***, Body temperature profiles. Top, Temporal changes in maximum body temperature of individual mice. Bottom, Group averages ±SEM (*N* = 5 per group). QIH(+) mice (blue) showed a marked drop in body temperature, maintained for over 24 h with additional CNO as needed, while no change was observed in the QIH(−) group (gray). ***D–G***, GFAP-positive cells around the injury site. Arrowheads indicate the insertion site of the fiber-optic cannula. QIH(−), ***D*** and ***E***; QIH(+), ***F*** and ***G***. Panels ***E*** and ***G*** are high-magnification views of the boxed regions in ***D*** and ***F***, respectively. ***H–K***, Iba1-positive cells around the injury site. QIH(−), ***H*** and ***I***; QIH(+), ***J*** and ***K***. Panels ***I*** and ***K*** are high-magnification views of the boxed regions in ***H*** and ***J***, respectively. ***L***, GFAP+ density in the 500 × 500 µm perilesional regions. GFAP+ density was reduced perilesional region of QIH(+) mice (unpaired two-tailed Student's *t* test, *t*_(8)_ = 6.23; *p* = 0.0003), but not contralaterally (*t*_(8)_ = 2.23; *p* = 0.056). ***M***, Iba1+ density in the same regions. Iba1+ density was reduced in both hemispheres (ipsilateral, *t*_(8)_ = 2.37; *p* = 0.045; contralateral, *t*_(8)_ = 5.41; *p* = 0.0006). **Abbreviations:** AVPe, anteroventral periventricular nucleus; mPFC, medial prefrontal cortex.

To capture potentially more informative glial changes, we opted to use a more subtle injury model that produces minimal damage and permits higher-resolution cellular analysis. Additionally, to determine whether the anti-inflammatory effects of QIH are consistent across brain regions, we chose the mPFC as a distinct target region (Fig. 4*A*–*C*). This model enabled high-resolution analysis of glial responses under tightly controlled stereotaxic conditions. Mice were perfusion-fixed 24.5 h after injury—corresponding to 24 h following QIH induction—and the brain tissue was collected for histological and molecular analyses.

We first assessed astrocytic and microglial activation using immunohistochemistry. GFAP immunostaining revealed dense accumulation of reactive astrocytes around the lesion site in mice of the QIH(−) group, whereas the QIH(+) group exhibited markedly reduced GFAP expression (Fig. 4*D*–*G*,*L*). Similarly, Iba1 staining showed significantly lower microglial density in the injured cortex of QIH(+) mice compared with the QIH(−) group (Fig. 3*H*–*K*,*M*), with a comparable reduction observed contralaterally, suggesting a systemic suppressive effect of QIH on glial activation.

Notably, in both the mPFC and striatal injury models of both QIH(−) and QIH(+) conditions, astrocytes formed a clearly defined GFAP-positive border immediately surrounding the lesion core, consistent with previous reports of reactive astrocyte-mediated scar formation (Sofroniew, 2015). However, the density of GFAP-positive astrocytes in the perilesional parenchyma outside this border was markedly reduced in the QIH(+) group compared with the QIH(−) group (Figs. 3*E*,*G*, 4*E*,*G*), indicating suppression of reactive gliosis beyond the immediate scar zone.

### Temporal dynamics of glial responses under QIH

**Figure 5.**
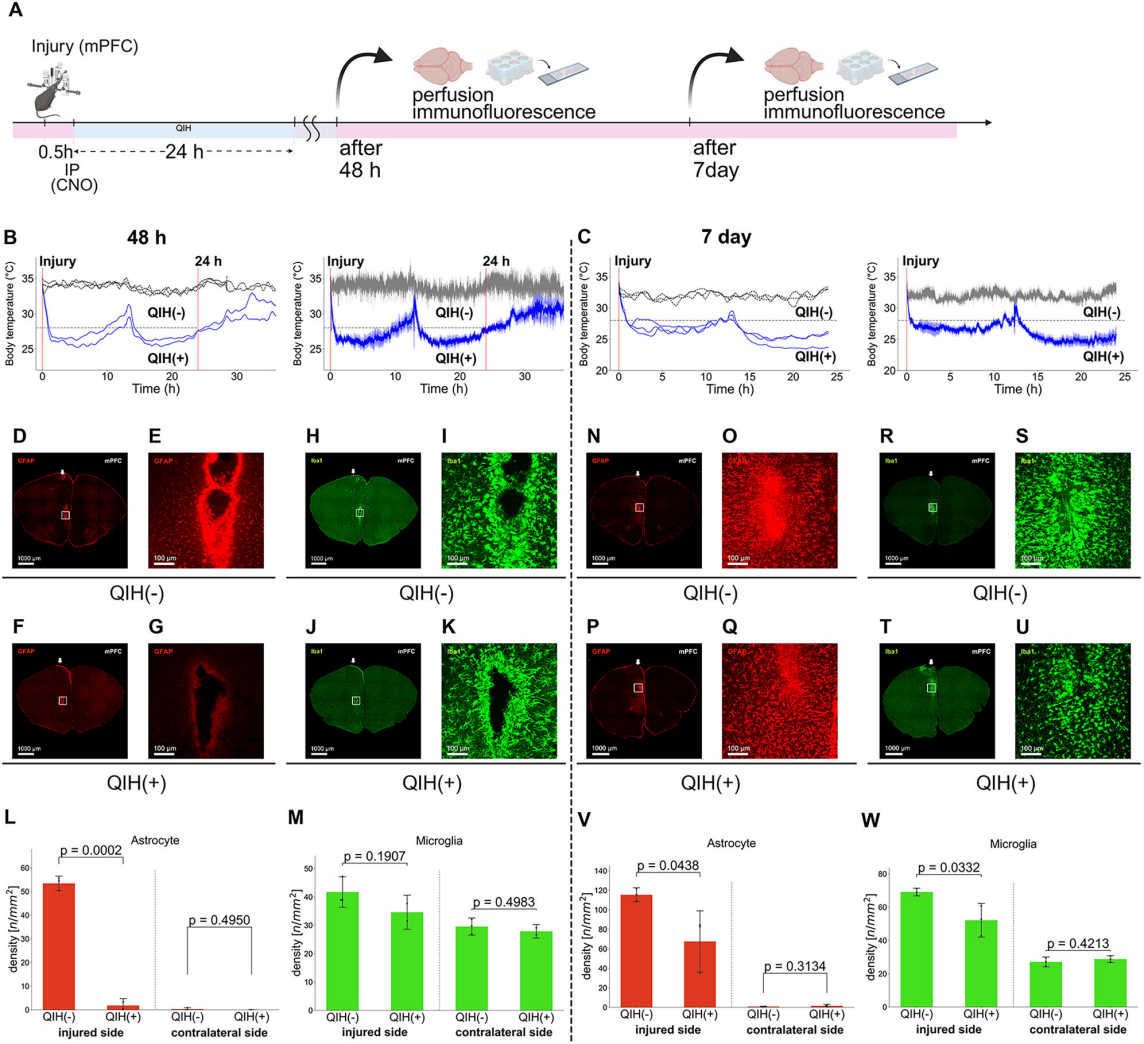
Effect of QIH on astrocytic and microglial responses in the mPFC at 48 h and 7 d after injury. ***A***, Experimental procedure. A stab injury was introduced into the mPFC using a fiber-optic cannula, followed by QIH induction. Brains were collected either 48 h or 7 d after injury for immunofluorescence analysis. ***B, C***, Body temperature profiles during QIH for 48 h (***B***) and 7 d (***C***) postinjury analysis. Left, Individual body temperatures. Right, Group-averaged temperature curves. Group averages ±SEM. QIH(+) mice (blue) showed a marked drop in body temperature, maintained for over 24 h with additional CNO as needed, while no change was observed in QIH(−) mice (gray). ***D–G***, Representative GFAP immunofluorescence images in the mPFC at 48 h postinjury. ***D, E***, QIH(−) group; (***F, G***) QIH(+) group. ***E, G***, High-magnification views of the rectangular regions in ***D*** and ***F***, respectively. ***H–K***, Representative Iba1 immunofluorescence images in the same region. ***H, I***, QIH(−) group; (***J, K***) QIH(+) group. ***I, K***, High-magnification views of the rectangular regions in ***H*** and ***J***, respectively. ***L***, Quantification of GFAP+ density in 500 × 500 µm perilesional regions. GFAP+ density was significantly reduced in perilesional resion of QIH(+) mice (unpaired two-tailed Student's *t* test, *t*_(3)_ = 22.47; *p* = 0.0002), but not contralateral side (*t*_(3)_ = 0.78; *p* = 0.495; *N* = 4 per group; QIH(+), *N* = 2; QIH(−), *N* = 3). ***M***, Quantification of Iba1+ density in the same regions. No significant differences were observed between QIH(−) and QIH(+) mice (injured side, *t*_(3)_ = 1.69; *p* = 0.191; contralateral side, *t*_(3)_ = 0.77; *p* = 0.498; QIH(−), *N* = 3; QIH(+), *N* = 2). ***N–Q***, Representative GFAP immunofluorescence images at 7 d postinjury. ***N, O***, QIH(−) group; (***P, Q***) QIH(+) group. ***O, Q***, High-magnification views of the rectangular regions in ***N*** and ***P***, respectively. ***R–U***, Representative Iba1 immunofluorescence images at 7 d postinjury. ***R, S***, QIH(−) group; (***T, U***) QIH(+) group. ***S, U***, High-magnification views of the rectangular regions in ***R*** and ***T***, respectively. ***V***, Quantification of GFAP+ density at 7 d. GFAP+ density was reduced in perilesional region of QIH(+) mice (*t*_(4)_ = 2.91; *p* = 0.044), but not contralateral side (*t*_(4)_ = −1.15; *p* = 0.313; *N* = 3 per group). ***W***, Quantification of Iba1+ density at 7 d. Iba1+ density was reduced in perilesional region of QIH(+) mice (*t*_(4)_ = 3.19; *p* = 0.033), but not contralaterall side(*t*_(4)_ = −0.90; *p* = 0.421). **Scale bars,** 1,000 µm (***D, F, H, J, N, P, R, T***), 100 µm (***E, G, I, K, O, Q, S, U***). **Abbreviations:** mPFC, medial prefrontal cortex; QIH, Q neuron-induced hypothermia.

To investigate the temporal dynamics of glial responses under QIH, we examined the perilesional mPFC at multiple time points after injury. At 24 h postinjury, QIH(+) mice exhibited a marked suppression of astrocytosis (Fig. 4). At 48 h, the QIH(+) group continued to show a strong reduction in reactive astrocytes, resembling the 24 h profile (Fig. 5*D*–*G*,*L*), whereas microglial numbers did not differ significantly between groups (Fig. 5*M*). By 7 d postinjury, astrocytosis in QIH(+) mice had increased but remained at lower levels compared with QIH(−) mice (Fig. 5*N*–*Q*,*V*). At this later time point, microglial numbers were also reduced in the QIH(+) group (Fig. 5*R–U,W*).

These findings indicate that QIH suppresses glial activation most prominently during the early postinjury phase. Importantly, this early suppression of astrocytic and microglial activation appears to result in sustained differences in the chronic phase, suggesting that QIH exerts its neuroprotective effects, at least in part, by modulating early inflammatory responses.

### QIH suppresses proinflammatory and phagocytic microglial markers

**Figure 6.**
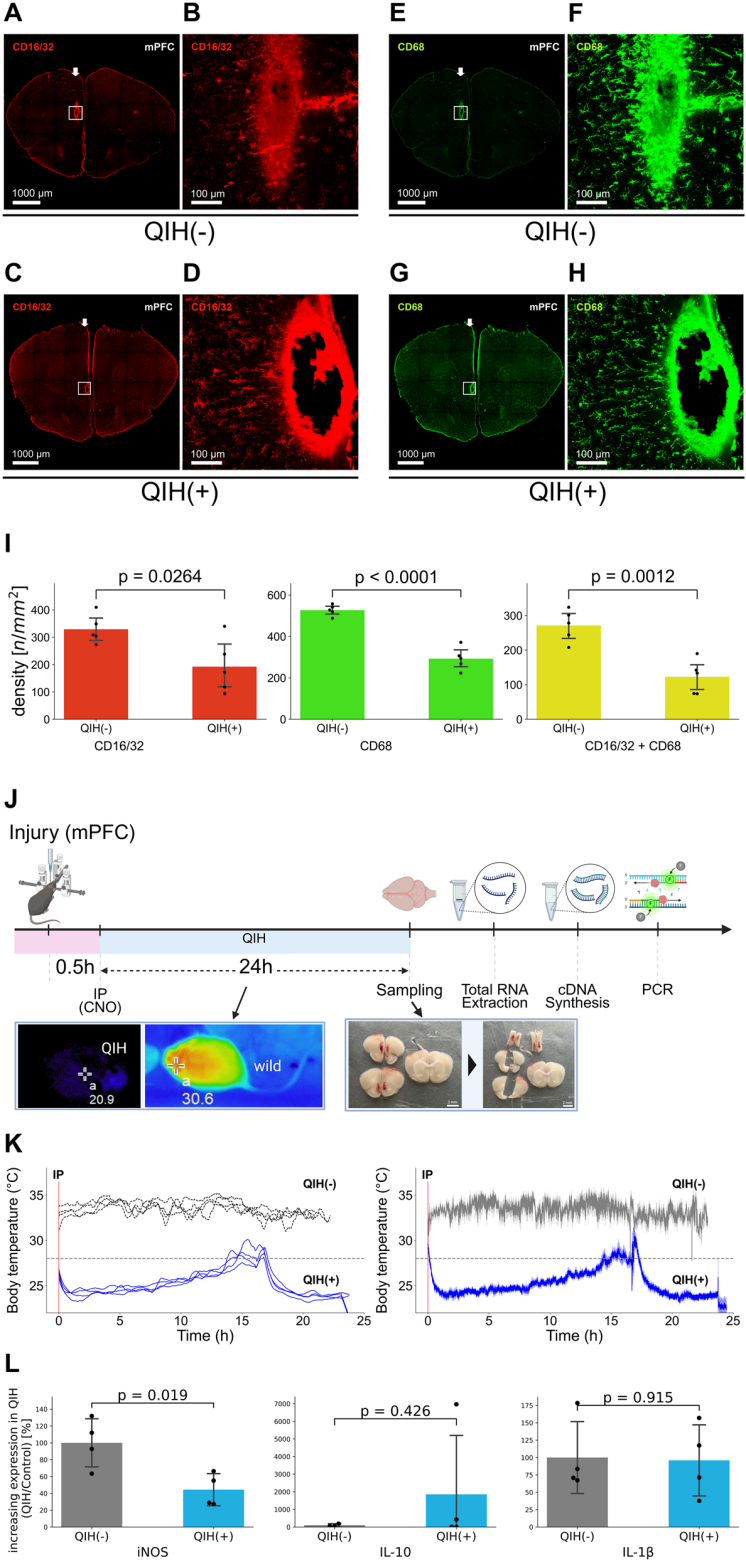
Effect of QIH on neuroinflammation around the injury site. ***A–D***, Expression of CD16/32 around the injury site in QIH(−) and QIH(+) groups. ***E–H***, Expression of CD68 around the injury site in QIH(−) and QIH(+) groups. ***I***, Density of CD16/32+ or CD68+ microglia around the injury site (*N* = 5 per group). Significant differences were observed between QIH(−) and QIH(+) groups in CD16/32 (unpaired two-tailed Student's *t* test, *t*_(8)_ = −2.72; *p* = 0.026), CD68 (*t*_(8)_ = −8.64; *p* < 0.0001), and double-positive cells (*t*_(8)_ = −4.92; *p* = 0.0012). ***J***, Schematic representation of the procedure for RT-qPCR analysis around the injury sites. ***K***, Left panel, Temporal changes in the maximum body temperature of each mouse. Right panel, Average ± SE of body temperature values shown in the left panel (*N* = 4 per group). QIH(+) mice (blue) showed sustained hypothermia (>24 h) with additional CNO, while QIH(−) mice (gray) maintained normothermia. ***L***, Expression levels of IL-1β, IL-10, and iNOS mRNAs in QIH(−) and QIH(+) groups (*N* = 4 per group). iNOS expression was significantly reduced in QIH(+) mice (*t*_(6)_ = 3.17; *p* = 0.019), whereas IL-10 (*t*_(5)_ = −0.87; *p* = 0.426) and IL-1β (*t*_(6)_ = 0.11; *p* = 0.915) did not differ significantly.

To further characterize microglial activation states, we examined the expression of CD16/32 (FcγRII/III) and CD68, markers associated with proinflammatory and phagocytic microglia, respectively. Both markers were significantly reduced in the injured cortex of mice in QIH(+) condition (Fig. 6*A*–*I*), indicating suppression of classically activated, M1-like microglial phenotypes.

We next assessed inflammatory gene expression by quantitative RT-PCR using the tissue from the perilesional mPFC collected at the same 24.5 h time point (Fig. 6*J*,*K*). Although expression levels of IL-1β and IL-10 did not differ significantly between groups, iNOS expression—a marker of oxidative and inflammatory stress—was significantly downregulated in mice of QIH(+) condition (Fig. 6*L*). This suggests that QIH selectively suppresses oxidative and phagocytic microglial activation while sparing broader cytokine responses.

Collectively, these findings demonstrate that QIH attenuates early neuroinflammation following cortical injury by suppressing glial activation and reducing the expression of proinflammatory and oxidative stress markers. This anti-inflammatory effect may contribute to the observed neuroprotection and functional recovery.

### QIH alters microglial morphology and functional states

**Figure 7.**
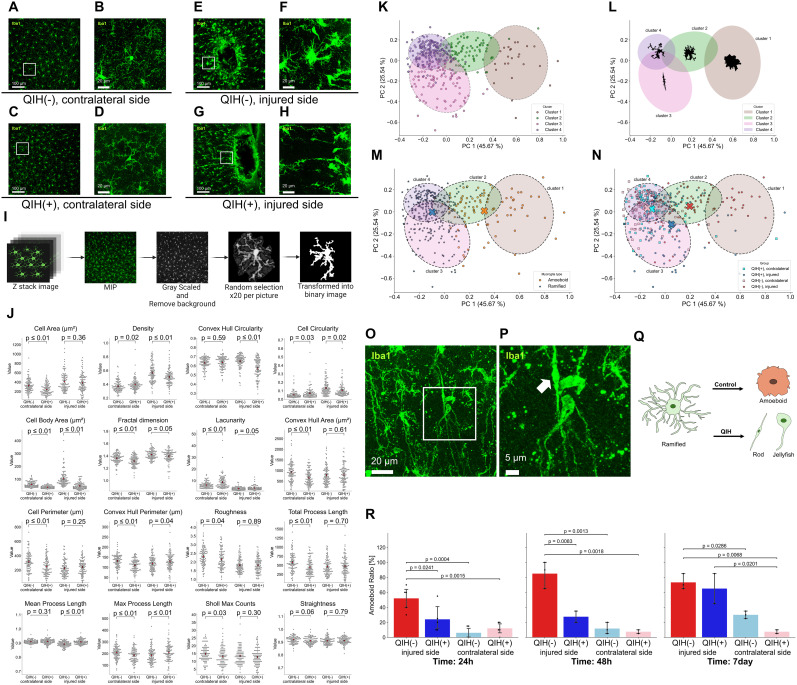
Morphological differences in microglia at the injury site in QIH-treated mice. ***A–H***, Representative images of microglia in the perilesional and contralateral mPFC regions under QIH(−) and QIH(+) conditions. Panels ***B, D, F***, and ***H*** show high-magnification views of the regions outlined in ***A***, ***C***, ***E***, and ***G***, respectively. Panels ***A–D*** display images from the contralateral side, while panels ***E–H*** show images from the injured side. ***I***, Strategy for microglial morphological analysis. ***J***, Comparison of morphological parameters between QIH(−) and QIH(+) groups (statistical parameters are provided in Table S1; *N* = 100 per group, 20 cells/mouse). Red dots indicate group means. Corresponding *p* values are shown in the figure. Here, we present all 16 parameters used for morphological analysis. ***K–N***, PCA revealed that microglia could be classified into four distinct clusters based on their morphological characteristics. ***K***, Microglia were classified into four clusters based on their morphological characteristics. ***L***, Representative schematic drawings of microglial morphology. ***M***, Distribution of microglia with amoeboid-like and ramified structures. X indicates the centroid of each group in the figure. ***N***, Distribution of microglia at the injured and contralateral sites under QIH(−) and QIH(+) groups. X indicates the centroid of each group in the figure. ***O***, Representative image of rod-shaped microglia observed in the perilesional cortex of the QIH(+) group. ***P***, A high-magnification view of the rectangular region shown in panel ***O***, highlighting the rod-like morphology of microglia. Arrows indicate rod-shaped microglia. ***Q***, Schematic illustration of microglial morphological changes after brain injury. Following injury, amoeboid microglia were predominantly observed in the QIH(−) group, whereas rod-shaped and jellyfish-like microglia were frequently found in the QIH(+) group. ***R***, Temporal changes in the proportion of amoeboid microglia in the samples. A two-way ANOVA revealed significant main effects of the group (*F*_(3,28)_ = 38.81; *p* < 0.001) and time (*F*_(2,28)_ = 8.02; *p* = 0.0018), as well as a significant interaction between the group and time (*F*_(6,28)_ = 2.84; *p* = 0.027).

To further elucidate how QIH modulates microglial behavior after brain injury, we conducted detailed morphometric analysis of Iba1-positive microglia in the mPFC at 24.5 h postinjury (Fig. 7*A*–*H*). Using a previously established morphometric pipeline (Fernández-Arjona et al., 2017; Green et al., 2022; Vidal-Itriago et al., 2022), high-resolution images of the injured cortex were processed for three-dimensional reconstruction and quantitative shape analysis using metrics such as process length, circularity, and solidity (Fig. 7*I*,*J*).

Hierarchical clustering and PCA based on these parameters identified four distinct microglial clusters (Fig. 7*K*). Microglia from the QIH(+) group were predominantly classified in Cluster 3, which displayed rod-like morphologies with elongated somata and aligned processes—structural features associated with surveillance or neuroprotective phenotypes. These cells have been implicated in neuroprotective roles following injury, potentially forming structural barriers around damaged neurons (Taylor et al., 2014). In contrast, the injured region in the QIH(−) group exhibited higher proportions of Cluster 1 microglia, which showed amoeboid-like forms consistent with inflammatory activation (Fig. 7*K*–*N*).

Overall morphometric distributions confirmed these patterns: microglia from injured sites of QIH(+) condition had significantly reduced circularity and increased process length compared with injured sites of QIH(−) condition (Fig. 7*J*; Table S1). The proportion of rod-shaped microglia in QIH(+) condition was especially elevated in regions adjacent to the lesion core (Fig. 7*O*–*Q*), where these cells often aligned parallel to the cortical surface and injury axis—a spatial pattern previously linked to tissue remodeling.

To extend this analysis, we also examined microglial morphology at 48 h and 7 d postinjury (Fig. S2; Tables S2 and S3). We calculated the percentage of microglia with an amoeboid index greater than the ramified index (defined as the “amoeboid ratio”) and presented these results in Figure 7*R*. Consistent with the 24 h findings, the QIH(+) group exhibited a significant reduction in the amoeboid ratio at 24 and 48 h compared with the QIH(−) group. By 7 d postinjury, however, this difference had disappeared. These observations indicate that QIH exerts its strongest modulatory effects on microglial dynamics within the first 48 h after injury, which likely contributes to the subsequent neuroprotective outcomes.

## Discussion

### QIH enhances functional recovery and preserves neuronal integrity after TBI

TBI initiates glial activation, leading to neuroinflammation, neuronal loss, and BBB disruption (Kreutzberg, 1996; Graeber and Streit, 2010; Muñoz-Ballester and Robel, 2023). Proinflammatory cytokines such as IL-1β and TNF-α amplify this response, perpetuating tissue damage (Arvin et al., 1996; Alam et al., 2020). Astrocytes become reactive, and microglia adopt amoeboid morphologies associated with neurotoxicity (Nimmerjahn et al., 2005; Hanisch and Kettenmann, 2007; Taylor and Sansing, 2013; Ben Haim and Rowitch, 2016). These activated microglia and astrocytes play a crucial role in TBI pathology, contributing to neurodegeneration and the progression of various CNS disorders (Gregersen et al., 2000; Block et al., 2007; Onwuekwe and Ezeala-Adikaibe, 2012; Kierdorf and Prinz, 2013; Mira et al., 2021).

We show that chemogenetically induced QIH promotes functional recovery and neuronal preservation in male mice with TBI. Using *Qrfp-iCre;Rosa26^dreaddm3^* mice, QIH was noninvasively induced and sustained for >24 h via repeated CNO injections. Though milder than viral systems, this hypothermia effectively conferred neuroprotection.

QIH(+) mice exhibited improved motor performance and grip strength after dorsal striatal stab injury, with increased neuronal density and reduced gliosis in the perilesional striatum (Figs. 1, 2). Notably, antigliotic effects persisted through POD 20, underscoring the therapeutic relevance of Q neuron activation for neurotrauma.

### Temporal dynamics of glial modulation by QIH

QIH modulates glial responses in a time-dependent manner after TBI. In the acute phase (24–48 h), QIH(+) mice showed markedly reduced perilesional gliosis, with fewer reactive astrocytes and a lower proportion of amoeboid microglia. Instead, microglia adopted rod-like morphologies, associated with neuroprotection and structural support, whereas QIH(−) mice exhibited classic inflammatory features.

By 7 d postinjury, glial differences between groups diminished (Figs. 5 and 7), though QIH(+) mice still showed attenuated gliosis at POD 20 (Fig. 2). These findings suggest that QIH primarily acts during the early inflammatory phase with lasting impact on the neuroinflammatory landscape.

This supports a model in which QIH suppresses initial neuroinflammation, mitigating secondary damage and promoting recovery—consistent with studies highlighting the importance of early glial modulation (Loane and Kumar, 2016; Witcher et al., 2021).

### QIH suppresses early glial activation and inflammatory responses

To investigate QIH-induced neuroprotection, we assessed glial responses after striatal and prefrontal injuries. QIH reduced GFAP and Iba1 immunoreactivity, indicating suppressed astrocytic and microglial activation (Figs. 3, 4). Notably, microglial changes were bilateral, suggesting a systemic anti-inflammatory effect (Figs. 3*M*, 4*M*).

QIH also decreased CD16/32, CD68, and iNOS expression, indicating reduced phagocytic activity and oxidative stress (Fig. 6*I*,*L*), while IL-1β and IL-10 levels remained unchanged, suggesting pathway-specific modulation.

Astrocytic scar borders near the lesion were preserved, but GFAP-positive astrocytes in the surrounding tissue were reduced (Figs. 3*G*, 4*G*, 5*G*). This indicates QIH suppresses astrocyte activation while maintaining scar integrity—protective against further damage (Sofroniew, 2015). Such region-specific modulation may limit secondary inflammation without compromising the protective role of the glial scar.

### Mechanisms underlying QIH-induced neuroprotection

A key mechanism by which QIH exerts neuroprotection may be through modulation of microglia–astrocyte interactions. Inflammatory microglia can induce neurotoxic A1 astrocytes (Witcher et al., 2018; Mira et al., 2021). QIH-treated mice exhibited attenuated astrocytic gliosis and decreased microglial activation, suggesting disruption of this detrimental loop. Although IL-1β and IL-10 mRNA levels did not differ significantly between QIH and control groups, iNOS expression was significantly reduced, indicating that QIH suppresses oxidative stress, potentially limiting ROS-mediated neuronal injury (Fig. 6*L*).

Microglia display remarkable morphological plasticity in response to environmental changes. In QIH(+) group microglia adopted ramified or rod-like shapes instead of amoeboid forms. Morphometric clustering revealed that rod-shaped microglia were enriched in QIH-treated animals, suggesting a shift toward a protective phenotype (Fig. 7). Rod microglia, which are typically absent from severely inflamed or damaged regions, are thought to support neuronal survival and structural integrity (Taylor et al., 2014). Their emergence under QIH(+) conditions, along with reduced iNOS expression, supports a link between antioxidative modulation and protective microglial remodeling (Roth et al., 2013).

QIH also suppressed markers of M1-like microglial polarization (CD16/32, CD68), consistent with a shift away from proinflammatory phenotypes. Though transcriptomic analysis was not performed, the observed morphologies are associated with reparative microglial states in prior studies. It is possible that QIH induces a gene expression program similar to that seen in hibernating mammals, characterized by anti-inflammatory and energy-conserving adaptations.

We observed clear attenuation of astrogliosis in the striatum 20 d after injury in the QIH(+) group compared with controls (Fig. 2). Nevertheless, a moderate level of gliosis persisted even under QIH conditions, suggesting that while early neuroinflammatory responses are suppressed, a delayed and controlled glial response still develops. This may reflect an initial suppression of A1-like astrocytes followed by delayed activation of A2-like, neuroprotective subtypes. This delayed gliosis may be beneficial, supporting repair while avoiding the damaging consequences of early, excessive inflammation. Indeed, controlled gliosis plays a role in neuroprotection, metabolic support, and scar formation (Bélanger and Magistretti, 2009; Shinozaki et al., 2017; Beylerli et al., 2024). Future work using glia-specific markers and single-cell sequencing would help elucidate these state transitions.

In addition, although we did not assess synaptic markers directly, the presence of rod-like microglia and improved behavior suggest maintenance of synaptic architecture. Future studies should assess synaptic integrity using markers such as synaptophysin or PSD-95 or functional imaging to confirm this hypothesis.

Although our findings demonstrate that QIH suppresses glial activation and neuroinflammation, the precise molecular mechanisms underlying these effects remain to be elucidated. Future studies using transcriptomic profiling, cell-type–specific manipulations, and pathway analyses will be essential to identify how Q neuron activation influences neuroimmune interactions. These efforts may clarify whether QIH acts via systemic metabolism, neuromodulation, or neuroendocrine signaling.

### QIH reprograms microglial morphology toward neuroprotective phenotypes

To further characterize the impact of QIH on microglial states, we performed high-dimensional morphometric profiling using a previously established pipeline (Taylor and Sansing, 2013). Hierarchical clustering based on shape parameters revealed four distinct microglial clusters. QIH-treated mice exhibited a predominance of Cluster 3 microglia, defined by rod-like morphologies with elongated somata and aligned processes (Fig. 7). These cells have been implicated in neuroprotective roles following injury, potentially forming structural barriers around damaged neurons (Taylor et al., 2014).

In contrast, the injury site of QIH(−) mice exhibited a predominance of amoeboid microglia—consistent with inflammatory activation. These morphological findings support the hypothesis that QIH not only reduces microglial activation but also promotes a shift toward a reparative, surveillance-like phenotype.

### Comparison to conventional hypothermia and clinical implications

Therapeutic hypothermia has long been investigated for neuroprotection in TBI, but its clinical utility remains limited by systemic side effects and technical challenges. QIH offers a novel solution by harnessing an endogenous neuronal mechanism to induce a torpor-like state without external cooling. This physiologically regulated hypothermia may offer enhanced stability and reduced risk compared with conventional methods.

While the neuroprotective effects observed in this study may be attributable to hypothermia itself, the means by which hypothermia is achieved is of critical relevance. In homeothermic animals such as mice, maintaining stable and prolonged hypothermia using external cooling typically necessitates deep anesthesia to suppress endogenous thermoregulatory defenses. However, in our experience and that of others, such procedures often result in severe physiological stress or mortality, making long-term external cooling an impractical or nonviable experimental condition.

In stark contrast, QIH represents an internally regulated and reversible hypometabolic state that enables mice to sustain deep hypothermia for over 24 h without the need for anesthesia or artificial cooling. All animals spontaneously rewarmed and survived, highlighting a significant advantage of QIH over conventional methods. This feature not only minimizes systemic complications but also makes it feasible to examine the effects of prolonged hypothermia in a physiologically stable and survival-compatible context. We believe this distinction offers important translational potential and justifies the use of QIH as a model for investigating endogenous neuroprotective pathways.

Our use of Qrfp-M3 mice allowed noninvasive, systemic induction of QIH. Although chemogenetic tools are not yet clinically deployable, the proof-of-concept established here opens avenues for pharmacological or neuromodulatory strategies targeting Q neurons or downstream effectors.

### Limitations and future directions

This study has several limitations. First, the effects of QIH were examined only in acute injury models; its efficacy in chronic or severe TBI remains to be tested. Second, while QIH clearly influenced microglial phenotype and inflammatory markers, its impact on long-term synaptic connectivity and cognitive outcomes is unknown. Third, the precise downstream mechanisms by which Q neurons modulate inflammation—whether via systemic metabolism, neuroimmune signaling, or neuroendocrine pathways—require further elucidation.

Future work should focus on delineating the molecular and circuit-level mechanisms underlying QIH-induced neuroprotection and evaluating the feasibility of QIH-mimetic interventions in translational settings.

In this study, we used only male mice to reduce variability. However, sex differences in neuroinflammatory responses and recovery after TBI are well documented. Future studies should examine whether QIH exerts similar neuroprotective effects in females.

### Conclusion

Our findings identify QIH as a novel and physiologically grounded neuroprotective state that promotes recovery after TBI. By reducing early glial activation, suppressing oxidative inflammation, and promoting neuroprotective microglial morphologies, QIH effectively interrupts secondary injury cascades. These results establish a foundation for future strategies that leverage neural control of metabolism to treat acute brain injury.
